# Clinical Effects of Photobiomodulation, Corticosteroids, and Nonsteroidal Anti‐Inflammatory Drugs Following Third Molar Surgery: A Systematic Review of Randomized Clinical Trials

**DOI:** 10.1155/ijod/5965550

**Published:** 2026-10-25

**Authors:** Érick Andres Alpaca Zevallos, Luciano Leocádio Teixeira Nogueira, Anderson Jara Ferreira, Camila Coelho Guimarães, Arthur Felipe de Brito Andrade, Ilan Hudson Gomes de Santana, Patrícia de Medeiros Loureiro Lopes, Elio Hitoshi Shinohara, Monique Danyelle Emiliano Batista Paiva

**Affiliations:** ^1^ University of Santa Maria Santa Maria Peru; ^2^ Federal University of Pelotas Pelotas Rio Grande do Sul Brazil ufpel.edu.br; ^3^ Federal University of Paraíba (UFPB) João Pessoa Paraíba Brazil ufpb.br; ^4^ São Leopoldo Mandic College João Pessoa Paraíba Brazil slmandic.edu.br; ^5^ Piracicaba Dental School University of Campinas Piracicaba São Paulo Brazil unicamp.br; ^6^ Regional Hospital of Osasco Osasco São Paulo Brazil

**Keywords:** corticosteroids, facial edema, nonsteroidal anti-inflammatory drugs, photobiomodulation, postoperative pain, third molar, trismus

## Abstract

**Objective:**

To systematically synthesize the effects and safety of corticosteroids, nonsteroidal anti‐inflammatory drugs (NSAIDs), and photobiomodulation, as evaluated within the comparisons available in randomized clinical trials, for controlling postoperative sequelae after third molar surgery.

**Methods:**

This systematic review followed PRISMA 2020 and was prospectively registered in PROSPERO (CRD420251178943). PubMed/MEDLINE, Embase, Scopus, Web of Science, and Google Scholar were searched without language or publication‐date restrictions. Randomized clinical trials evaluating at least one eligible intervention and reporting pain, edema, trismus, rescue medication, or adverse events were included. Risk of bias was assessed with Cochrane RoB 2, and a structured narrative synthesis was performed by intervention class, comparator type, outcome, and follow‐up period.

**Results:**

Thirty‐six randomized trials involving 1838 participants were included: 13 primarily evaluated corticosteroids, 11 NSAIDs, 7 photobiomodulation, and 5 direct or combined therapeutic comparisons. Twenty‐seven trials were judged at low risk of bias, seven raised some concerns, and two were at high risk. Corticosteroids were frequently associated with reductions in edema and trismus within placebo‐controlled and active‐comparator trials; NSAIDs showed more consistent effects on pain than on edema or trismus; and photobiomodulation produced heterogeneous, protocol‐dependent findings. Only a small number of trials directly compared different treatment classes. Substantial clinical and methodological heterogeneity precluded meta‐analysis and a valid indirect ranking.

**Conclusion:**

The three approaches may improve different postoperative outcomes, but the available evidence does not support a definitive hierarchy among them. Direct head‐to‐head trials with standardized outcomes, treatment protocols, and follow‐up periods are required.

## 1. Introduction

The surgical removal of impacted or partially impacted third molars is among the most frequent procedures in oral and maxillofacial surgery and is commonly followed by acute inflammatory sequelae, particularly pain, facial edema, and trismus [1–3]. These outcomes may impair oral intake, speech, oral hygiene, sleep, and return to usual activities, making postoperative symptom control an important component of patient‐centered surgical care [4, 5].

Postoperative pain is driven by tissue injury, peripheral nociceptor sensitization, and the release of prostaglandins and proinflammatory cytokines. Edema results from vasodilation and increased vascular permeability, whereas trismus is related to inflammation and protective muscle spasm involving the masticatory muscles and adjacent fascial tissues [6–8]. The magnitude and duration of these manifestations are influenced by surgical difficulty, operative time, tissue manipulation, anatomical characteristics, and patient‐related factors.

NSAIDs are widely prescribed because inhibition of cyclooxygenase reduces prostaglandin‐mediated hyperalgesia and inflammation. Corticosteroids act more proximally in the inflammatory cascade through phospholipase A2 inhibition and broader modulation of inflammatory mediators, which may be particularly relevant for edema and functional limitation. Photobiomodulation has also been investigated as a nonpharmacological strategy capable of modulating mitochondrial activity, inflammatory signaling, microcirculation, and nociceptive processing without systemic drug exposure [9, 10].

However, the evidence base is characterized by substantial heterogeneity in corticosteroid dose and route, NSAID selection and timing, laser wavelength and dosimetry, comparator type, outcome measurement, and follow‐up schedule. Most trials evaluate one intervention against placebo, sham treatment, another dose or route, or a single active comparator; relatively few directly compare distinct treatment classes. Therefore, cross‐treatment conclusions should not be interpreted as a three‐way comparative ranking unless supported by direct evidence or a connected evidence network.

This systematic review aimed to synthesize the effects and safety of corticosteroids, NSAIDs, and photobiomodulation for pain, edema, trismus, rescue medication consumption, and adverse events after third molar surgery. The synthesis was structured by intervention class and comparator type, with direct comparisons presented separately from within‐class and placebo‐controlled evidence.

## 2. Methods

### 2.1. Study Design and Registration

This systematic review was conducted in accordance with Cochrane methodological principles and reported according to the PRISMA 2020 statement [11]. The protocol was prospectively registered in the International Prospective Register of Systematic Reviews (PROSPERO; CRD420251178943) [12]. Ethical approval was not required because the review used data from previously published studies.

The review question was structured using the PICO framework. The population comprised adolescents or adults undergoing surgical removal of impacted or partially impacted third molars. Eligible interventions included systemic or locally administered corticosteroids, NSAIDs, and photobiomodulation. Eligible comparators included placebo, sham irradiation, no additional intervention, standard postoperative care, another dose or route of the same intervention, another agent within the same pharmacological class, or an active intervention from a different class. Outcomes of interest were postoperative pain, facial edema, trismus or maximum mouth opening, rescue analgesic consumption, adverse events, and patient‐reported recovery outcomes.

### 2.2. Information Sources and Search Strategy

A comprehensive electronic search was performed in PubMed/MEDLINE, Embase, Scopus, Web of Science, and Google Scholar without restrictions on the publication date or language. Controlled vocabulary and free‐text terms for third molar surgery were combined with terms for corticosteroids, NSAIDs, and photobiomodulation, as well as postoperative pain, edema, swelling, and trismus. The intervention blocks were connected with the Boolean operator OR so that studies evaluating any eligible modality could be retrieved. Reference lists of eligible studies were also screened. Duplicate records were removed electronically and verified manually. The complete database‐specific search strategies are presented in Table 1.

**Table 1:** Database‐specific search strategies.

Database	Search strategy
PubMed/MEDLINE	(“Third Molar”[Mesh] OR “third molar ^∗^”[tiab] OR “wisdom tooth”[tiab] OR “wisdom teeth”[tiab]) AND ((“Low‐Level Light Therapy”[Mesh] OR photobiomodulation[tiab] OR “low‐level laser ^∗^”[tiab] OR LLLT[tiab]) OR (“Anti‐Inflammatory Agents, Non‐Steroidal”[Mesh] OR NSAID ^∗^[tiab] OR ibuprofen[tiab] OR diclofenac[tiab] OR ketoprofen[tiab] OR dexketoprofen[tiab] OR naproxen[tiab] OR meloxicam[tiab] OR etodolac[tiab] OR etoricoxib[tiab] OR celecoxib[tiab] OR nimesulide[tiab]) OR (“Adrenal Cortex Hormones”[Mesh] OR corticosteroid ^∗^[tiab] OR dexamethasone[tiab] OR betamethasone[tiab] OR methylprednisolone[tiab] OR prednisolone[tiab])) AND (“Postoperative Pain”[Mesh] OR pain[tiab] OR edema[tiab] OR oedema[tiab] OR swelling[tiab] OR trismus[tiab]) AND (randomized controlled trial[pt] OR controlled clinical trial[pt] OR random ^∗^[tiab] OR placebo[tiab] OR trial[tiab])
Embase	(‘third molar’/exp OR ‘third molar ^∗^’:ti,ab OR ‘wisdom tooth’:ti,ab OR ‘wisdom teeth’:ti,ab) AND ((‘low level laser therapy’/exp OR ‘photobiomodulation’/exp OR ‘low‐level laser ^∗^’:ti,ab OR LLLT:ti,ab) OR (‘nonsteroidal antiinflammatory agent’/exp OR NSAID ^∗^:ti,ab OR ibuprofen:ti,ab OR diclofenac:ti,ab OR ketoprofen:ti,ab OR naproxen:ti,ab OR meloxicam:ti,ab OR etodolac:ti,ab OR etoricoxib:ti,ab OR celecoxib:ti,ab OR nimesulide:ti,ab) OR (‘corticosteroid’/exp OR corticosteroid ^∗^:ti,ab OR dexamethasone:ti,ab OR betamethasone:ti,ab OR methylprednisolone:ti,ab OR prednisolone:ti,ab)) AND (‘postoperative pain’/exp OR pain:ti,ab OR edema:ti,ab OR oedema:ti,ab OR swelling:ti,ab OR trismus:ti,ab) AND (‘randomized controlled trial’/exp OR random ^∗^:ti,ab OR placebo:ti,ab OR trial:ti,ab)
Scopus	TITLE‐ABS‐KEY(“third molar ^∗^” OR “wisdom tooth” OR “wisdom teeth”) AND TITLE‐ABS‐KEY((photobiomodulation OR “low‐level laser ^∗^” OR LLLT) OR (NSAID ^∗^ OR ibuprofen OR diclofenac OR ketoprofen OR dexketoprofen OR naproxen OR meloxicam OR etodolac OR etoricoxib OR celecoxib OR nimesulide) OR (corticosteroid ^∗^ OR dexamethasone OR betamethasone OR methylprednisolone OR prednisolone)) AND TITLE‐ABS‐KEY(pain OR edema OR oedema OR swelling OR trismus) AND TITLE‐ABS‐KEY(random ^∗^ OR placebo OR trial)
Web of Science	TS = (“third molar ^∗^” OR “wisdom tooth” OR “wisdom teeth”) AND (photobiomodulation OR “low‐level laser ^∗^” OR LLLT) OR (NSAID ^∗^ OR ibuprofen OR diclofenac OR ketoprofen OR dexketoprofen OR naproxen OR meloxicam OR etodolac OR etoricoxib OR celecoxib OR nimesulide) OR (corticosteroid ^∗^ OR dexamethasoneOR betamethasone OR methylprednisolone OR prednisolone) AND (pain OR edema OR oedema OR swelling OR trismus) AND (random ^∗^ OR placebo OR trial)
Google Scholar	“third molar” (photobiomodulation OR “low‐level laser” OR corticosteroid OR dexamethasone OR NSAID OR ibuprofen OR diclofenac) (pain OR edema OR swelling OR trismus) randomized trial

*Note:* The asterisk ( ^∗^) denotes that the search syntax was adapted according to the specific indexing terms, controlled vocabulary, and search functionalities of each database.

### 2.3. Eligibility Criteria

Randomized parallel‐group, split‐mouth, crossover, or placebo‐controlled clinical trials in humans were eligible when they evaluated at least one intervention of interest following third molar surgery, had a minimum postoperative follow‐up of 24 h, and reported at least one prespecified outcome. Trials comparing doses, routes, administration times, agents within the same class, active treatments from different classes, or combination regimens were eligible. Studies were excluded if they were nonrandomized, observational, animal or in vitro investigations, case reports or case series, secondary publications without usable primary data, or reports in which the effect of an eligible intervention could not be distinguished from concomitant therapy.

### 2.4. Study Selection

Two reviewers independently screened titles and abstracts and subsequently assessed potentially eligible full‐text reports. Disagreements were resolved by discussion and, when necessary, consultation with a third reviewer. The reasons for full‐text exclusion were recorded. The study‐selection process was summarized in a PRISMA 2020 flow diagram.

### 2.5. Data Extraction

Two reviewers independently extracted study year and country, design, sample size, participant characteristics, surgical characteristics, intervention and comparator details, corticosteroid or NSAID dose and route, photobiomodulation wavelength and dosimetry, timing and number of sessions, outcome definitions, assessment time points, numerical results, rescue medication use, adverse events, and authors’ conclusions. Discrepancies were resolved by consensus. When required data were incompletely reported, the corresponding authors were contacted.

### 2.6. Risk of Bias Assessment

The risk of bias was assessed independently by two reviewers using the Cochrane Risk of Bias 2 tool for the primary outcome of postoperative pain. The five domains were the randomization process, deviations from intended interventions, missing outcome data, measurement of the outcome, and selection of the reported result. Domain‐level and overall judgments were categorized as low risk, some concerns, or high risk. Disagreements were resolved by consensus.

### 2.7. Data Synthesis and Assessment of Meta‐Analysis Feasibility

A structured narrative synthesis was planned because the eligible trials were expected to differ in intervention protocols and comparator architecture. Studies were first grouped into four categories: corticosteroid trials, NSAID trials, photobiomodulation trials, and direct or combined therapeutic comparisons. Within each category, studies were further organized by comparator type: placebo or sham, standard care, dose or route comparison, within‐class active comparison, between‐class active comparison, or combination therapy. Findings were synthesized separately for pain, edema, trismus, rescue medication consumption, and adverse events, taking into account direction of effect, statistical significance, follow‐up period, and risk of bias.

Meta‐analysis was considered only when at least two trials reported sufficiently similar intervention contrasts, outcome definitions, follow‐up times, and summary statistics. Network meta‐analysis was not undertaken because the evidence network was sparse and disconnected, with limited common comparators across treatment classes. No indirect comparison was used to establish a hierarchy among corticosteroids, NSAIDs, and photobiomodulation.

## 3. Results

### 3.1. Study Selection and Characteristics

The searches identified 1593 records. After the removal of 556 duplicates, 1037 records were screened. Sixty‐nine reports were sought for retrieval, four could not be obtained, and 65 full‐text reports were assessed. Twenty‐nine reports were excluded, leaving 36 randomized clinical trials for qualitative synthesis (Figure 1).

**Figure 1:**
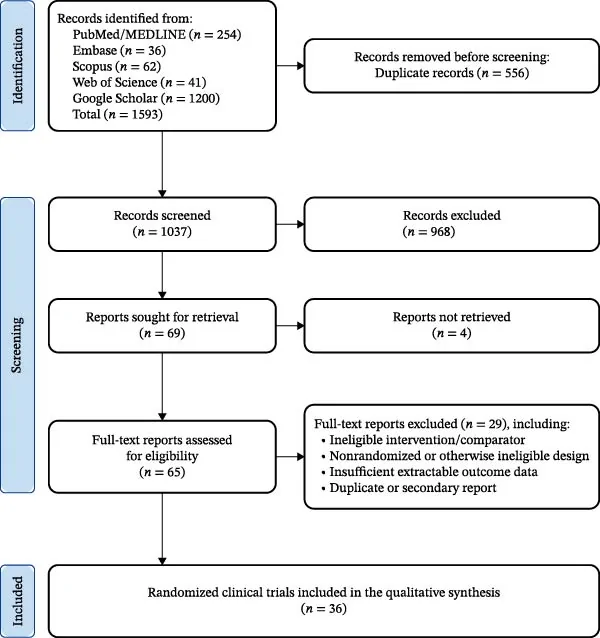
PRISMA 2020 flow diagram of study identification, screening, eligibility, and inclusion.

The 36 trials were published between 2006 and 2025 and included 1838 participants. Sample sizes ranged from 11 to 195. Most studies enrolled systemically healthy young adults undergoing mandibular third molar extraction and used either parallel‐group or split‐mouth designs. Thirteen trials primarily evaluated corticosteroids, 11 evaluated NSAIDs, seven evaluated photobiomodulation, and five evaluated direct between‐class or combined therapeutic strategies. The included interventions varied considerably in dose, route, timing, laser wavelength, irradiance, energy density, number of sessions, and postoperative follow‐up. Detailed study characteristics, comparator architecture, and main findings are summarized in Table 2.

**Table 2:** Characteristics, comparator architecture, and main findings of the included randomized clinical trials.

Class	Study	Country/year	Design, *n*	Intervention	Comparator	Outcomes	Main findings
Corticosteroid	Latt et al. [13]	Thailand, 2016	Randomized, prospective, split‐mouth clinical trial; *n* = 31	Dexamethasone (8 mg) via pterygomandibular space	Contralateral control/placebo	Pain, edema, and trismus; rescue medication or quality of life when reported	Significant difference in VAS scores on the day of operation and 1st postoperative day (*p* < 0.05).
Corticosteroid	Priyanga et al.[14]	India, 2022	Prospective randomized controlled study; *n* = 150	Dexamethasone (8 mg) sublingual vs. intramuscular	Intramuscular dexamethasone (8 mg)	Pain, edema, and trismus; rescue medication or quality of life when reported	Sublingual dexamethasone produced lower pain scores and greater mouth opening than intramuscular administration on days 1, 3, and 7.
Corticosteroid	Marques et al. [15]	Brazil, 2022	Randomized, double‐blind, split‐mouth trial; *n* = 22	Dexamethasone (4 mg) intra‐alveolar vs. oral	Oral dexamethasone (4 mg)	Pain, edema, and trismus; rescue medication or quality of life when reported	No significant differences between oral and intra‐alveolar routes for pain, edema, or trismus (*p* > 0.05).
Corticosteroid	Ibikunle et al. [16]	Nigeria, 2016	Randomized controlled trial; *n* = 186	Prednisolone (40 mg) submucosal vs. oral	Oral prednisolone (40 mg)	Pain, edema, and trismus; rescue medication or quality of life when reported	Submucosal group showed overall lower values for pain and swelling compared to oral prednisolone.
Corticosteroid	Chaudhary et al. [17]	India, 2015	Prospective, double‐blind and randomized study; *n* = 195	Dexamethasone (4 mg IV vs. 8 mg oral)	Intravenous vs. oral dexamethasone	Pain, edema, and trismus; rescue medication or quality of life when reported	No significant difference in swelling, trismus, or pain between the two routes (*p* > 0.05).
Corticosteroid	Poorna et al. [18]	India, 2024	Split‐mouth randomized double‐blind study; *n* = 35	Dexamethasone (8 mg) mixed with 2% lignocaine	Lignocaine with adrenaline alone	Pain, edema, and trismus; rescue medication or quality of life when reported	Faster onset (69 s) and longer duration of anesthesia (+128 min, *p* < 0.001); significantly less pain.
Corticosteroid	Graziani et al. [19]	Italy, 2006	Split‐mouth randomized double‐masked trial; *n* = 43	Dexamethasone (4/10 mg endo‐alveolar; 4 mg submucosal)	Contralateral control/placebo	Pain, edema, and trismus; rescue medication or quality of life when reported	All steroid‐treated groups showed reductions in edema and trismus compared to control.
Corticosteroid	Kaewkumnert et al. [20]	Thailand, 2020	Randomized, triple‐blind, split‐mouth trial; *n* = 27	Dexamethasone (4 mg) intraosseous vs. submucosal	Intraosseous vs. submucosal dexamethasone	Pain, edema, and trismus; rescue medication or quality of life when reported	No difference in pain or swelling; however, trismus on day 3 was significantly better in the SM group.
Corticosteroid	Marques et al. [21]	Spain, 2014	Split‐mouth, triple‐blind, randomized trial; *n* = 25	Betamethasone (12 mg) submucosal	Placebo	Pain, edema, and trismus; rescue medication or quality of life when reported	No significant differences between betamethasone and placebo for pain, swelling, or trismus.
Corticosteroid	Larsen et al. [22]	Denmark, 2021	Double‐blind, split‐mouth, randomized controlled trial; *n* = 52	Methylprednisolone (20 mg, 30 mg, 40 mg IM)	Placebo and alternative methylprednisolone doses	Pain, edema, and trismus; rescue medication or quality of life when reported	Methylprednisolone revealed no significant differences in pain or trismus compared with placebo.
Corticosteroid	Mojsa et al. [23]	Poland, 2017	Prospective, randomized, double‐blind trial; *n* = 90	Dexamethasone (4 mg) submucosal (before vs. after)	Placebo and preop vs. postop timing	Pain, edema, and trismus; rescue medication or quality of life when reported	Postoperative injection provided better pain control than preoperative injection.
Corticosteroid	Srivastava et al. [24]	India, 2021	Split‐mouth, randomized, triple‐blind trial; *n* = 20	Dexamethasone (8 mg) vs. methylprednisolone (40 mg) IM	Methylprednisolone (40 mg)	Pain, edema, and trismus; rescue medication or quality of life when reported	Dexamethasone showed better day‐2 postoperative outcomes than methylprednisolone in this small split‐mouth trial.
Corticosteroid	Vyas et al. [25]	India, 2014	Randomized controlled trial; *n* = 60	Methylprednisolone acetate (40 mg) preop vs. postop	Postoperative methylprednisolone	Pain, edema, and trismus; rescue medication or quality of life when reported	Preoperative administration showed superior results in oral aperture, pain, and swelling (*p* < 0.05).
NSAID	Ramos et al. [26]	Brazil, 2022	Randomized, double‐blind, split‐mouth trial; *n* = 21	Ibuprofen–arginine (600 mg + 555 mg)	Ibuprofen alone	Pain, edema, and trismus; rescue medication or quality of life when reported	Significant differences in facial edema (*p* < 0.05); lower number of rescue analgesics in the arginine group.
NSAID	Gülnahar and Kupeli [27]	Turkey, 2018	Randomized controlled study; *n* = 60	Intravenous ibuprofen (800 mg)	Control/standard postoperative regimen	Pain, edema, and trismus; rescue medication or quality of life when reported	Significant reduction in trismus and edema in the IV ibuprofen group compared to controls (*p* < 0.05).
NSAID	Pérez‐González et al. [28]	Spain, 2023	Crossover randomized controlled trial; *n* = 11	Ibuprofen (400 mg) (Chronotherapy)	Alternative ibuprofen administration schedules	Pain, edema, and trismus; rescue medication or quality of life when reported	Alternative ibuprofen timing schedules did not significantly differ for postoperative pain or swelling.
NSAID	Aznar‐Arasa et al. [29]	Spain, 2012	Triple‐blind, randomized controlled trial; *n* = 109	Ibuprofen (600 mg) preop vs. postop	Postoperative ibuprofen	Pain, edema, and trismus; rescue medication or quality of life when reported	No significant differences regarding pain, rescue analgesics, swelling, or trismus (*p* > 0.05).
NSAID	Calvo et al. [30]	Brazil, 2007	Double‐blind, randomized, crossover study; *n* = 49	Meloxicam (7.5 mg vs. 15 mg)	Meloxicam 7.5 mg vs. 15 mg	Pain, edema, and trismus; rescue medication or quality of life when reported	Meloxicam 15 mg provided better pain control than 7.5 mg in procedures requiring osteotomy; differences were less apparent in less traumatic cases.
NSAID	Costa et al. [31]	Brazil, 2015	Split‐mouth, randomized, triple‐blind trial; *n* = 18	Etoricoxib (120 mg)	Placebo	Pain, edema, and trismus; rescue medication or quality of life when reported	Significantly reduced pain scores (*p* < 0.001) and lower rescue medication consumption.
NSAID	Bassyoni [32]	Saudi Arabia, 2024	Double‐blinded randomized controlled trial; *n* = 90	Celecoxib (100 mg), diclofenac (50 mg), ibuprofen (400 mg)	Celecoxib vs. diclofenac vs. ibuprofen	Pain, edema, and trismus; rescue medication or quality of life when reported	Celecoxib and diclofenac provided better pain control than ibuprofen; between‐group differences for edema and trismus were less consistent.
NSAID	Kara et al. [33]	Turkey, 2010	Randomized, double‐blind, crossover trial; *n* = 30	Oxaprozin (1200 mg) vs. naproxen sodium (550 mg)	Oxaprozin, naproxen, and placebo	Pain, edema, and trismus; rescue medication or quality of life when reported	Both drugs superior to placebo for pain (*p* ≤ 0.05) but no significant effect on facial swelling.
NSAID	Akbulut et al. [34]	Turkey, 2014	Randomized, double‐blind, crossover trial; *n* = 42	Diclofenac potassium (50 mg), etodolac (200 mg), naproxen (275 mg)	Naproxen, etodolac, and diclofenac	Pain, edema, and trismus; rescue medication or quality of life when reported	Swelling on the 2nd day was significantly lowest with diclofenac potassium (*p* = 0.027).
NSAID	Pouchain et al. [35]	Brazil, 2015	Split‐mouth, prospective, randomized, double‐blind trial; *n* = 18	Nimesulide (100 mg) vs. ketoprofen (100 mg)	Nimesulide vs. ketoprofen	Pain, edema, and trismus; rescue medication or quality of life when reported	Peak pain at 6 h (nimesulide) and 12 h (ketoprofen); no significant difference between groups.
NSAID	Silva de Oliveira et al. [36]	Brazil, 2016	Randomized clinical trial, paired crossover; *n* = 20	Ibuprofen (600 mg) vs. etodolac (300 mg)	Ibuprofen vs. etodolac	Pain, edema, and trismus; rescue medication or quality of life when reported	Etodolac group had significantly less edema and trismus at 48 h (*p* < 0.05).
Photobiomodulation	Camolesi et al. [37]	Spain, 2025	Randomized, double‐blind, split‐mouth trial; *n* = 83	Ga–Al–As diode laser (808 nm, 100 mW)	Sham irradiation (split‐mouth)	Pain, edema, and trismus; rescue medication or quality of life when reported	Significant reduction in pain at all times (*p* < 0.01); reductions in edema and trismus on days 2 and 7 (*p* < 0.01).
Photobiomodulation	Momeni et al. [38]	Iran, 2021	Double‐blind randomized clinical trial; *n* = 25	Intraoral laser diode (940 nm, 0.5 W)	Sham irradiation	Pain, edema, and trismus; rescue medication or quality of life when reported	Pain was significantly lower in the intervention group on the 6th and 7th days (*p* < 0.05).
Photobiomodulation	Raiesian et al. [39]	Iran, 2017	Prospective, randomized double‐blind study; *n* = 44	Diode laser (980 nm, 0.3 W)	Sham irradiation/standard pharmacotherapy	Pain, edema, and trismus; rescue medication or quality of life when reported	Mean pain scores at 24 h and 1 week were significantly lower in the laser group (*p* < 0.05).
Photobiomodulation	Cetira Filho et al. [40]	Brazil, 2022	Randomized, split‐mouth, triple‐blind trial; *n* = 40	LLLT (Laser 100 mW) + nimesulide (100 mg)	Nimesulide with sham/no active PBM	Pain, edema, and trismus; rescue medication or quality of life when reported	Active PBM was associated with lower early pain and reduced edema compared with the condition without active PBM; all participants received nimesulide.
Photobiomodulation	Sigaroodi et al. [41]	Iran, 2023	Double‐blind randomized clinical trial, split‐mouth; *n* = 32	Ga–Al–As diode laser (808 nm, 200 mW)	Sham irradiation	Pain, edema, and trismus; rescue medication or quality of life when reported	Mean pain scores were significantly lower in the laser group (*p* < 0.001); swelling and mouth opening improved.
Photobiomodulation	Ferreira et al. [42]	Brazil, 2022	Randomized, double‐blind, crossover trial; *n* = 21	Laser (660 nm/789 nm)	Alternative single‐ and multiple‐session PBM protocols	Pain, edema, and trismus; rescue medication or quality of life when reported	No statistical differences for pain, swelling (*p* = 0.958), or trismus (*p* = 0.837) between the two protocols.
Photobiomodulation	Momeni et al. [43]	Iran, 2022	Split‐mouth, randomized, placebo‐controlled trial; *n* = 25	Extraoral diode laser (940 nm, 10 J/cm^2^)	Sham irradiation	Pain, edema, and trismus; rescue medication or quality of life when reported	Mean pain score was significantly lower in the laser group (*p* < 0.05); edema was not significant.
Direct/combined comparison	Ramires et al. [44]	Brazil, 2021	Randomized, triple‐blind, crossover trial; *n* = 40	Dexamethasone (8 mg) + etodolac (300 mg)	Dexamethasone alone and etodolac alone	Pain, edema, and trismus; rescue medication or quality of life when reported	VAS scores and number of rescue analgesics were lower in the DEX + ETO group (*p* < 0.001).
Direct/combined comparison	Lima et al. [45]	Brazil, 2017	Randomized, double‐blind, split‐mouth trial; *n* = 30	Dexamethasone (4 mg) vs. diclofenac sodium (50 mg)	Diclofenac sodium (50 mg)	Pain, edema, and trismus; rescue medication or quality of life when reported	Mouth opening (*p* < 0.05) and swelling (*p* < 0.05) were significantly better with dexamethasone.
Direct/combined comparison	Lima et al. [46]	Brazil, 2018	Split‐mouth randomized triple‐blind trial; *n* = 15	Dexamethasone (8 mg) vs. diclofenac (50 mg) + codeine (50 mg)	Diclofenac plus codeine	Pain, edema, and trismus; rescue medication or quality of life when reported	Dexamethasone produced lower early pain and numerically less edema than diclofenac plus codeine; the edema result was not statistically significant (*p* = 0.08).
Direct/combined comparison	Rodrigues et al. [47]	Brazil, 2019	Prospective, randomized, split‐mouth trial; *n* = 19	Dexamethasone (4 mg IM) vs. etoricoxib (90 mg oral)	Etoricoxib (90 mg)	Pain, edema, and trismus; rescue medication or quality of life when reported	Dexamethasone reduced edema in selected facial measurements at 72 h compared with etoricoxib; other outcomes were not consistently different.
Direct/combined comparison	Momesso et al. [48]	Brazil, 2021	Triple‐blind randomized clinical trial; *n* = 60	Dexamethasone (8 mg) alone or + NSAIDs	Dexamethasone alone and dexamethasone–NSAID combinations	Pain, edema, and trismus; rescue medication or quality of life when reported	DEX + ETO and DEX + KET significantly decreased pain perception at 6 h.

### 3.2. Risk of Bias

For postoperative pain, 27 trials were judged to have a low overall risk of bias, seven raised some concerns, and two were classified as high risk (Figure 2). The most frequent concerns involved incomplete reporting of sequence generation or allocation concealment, difficulties with blinding in photobiomodulation or route‐comparison trials, and limited information about prespecified analyses. Missing outcome data were generally well managed. The two high‐risk judgments reflected multiple concerns involving randomization, outcome measurement, and selective reporting. These limitations were considered when interpreting the direction and consistency of the effects.

**Figure 2:**
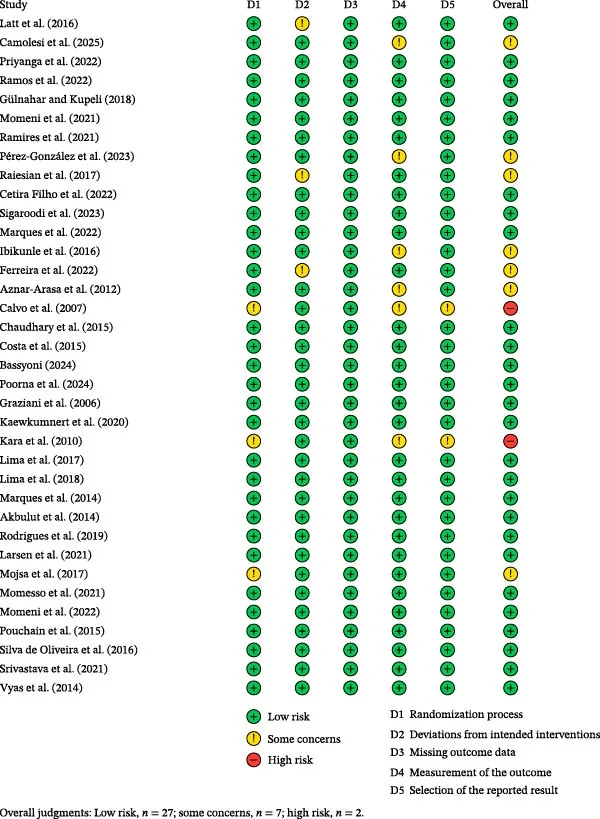
Risk of bias assessment for postoperative pain using the Cochrane risk of bias 2 tool.

### 3.3. Corticosteroid Trials

Thirteen trials primarily evaluated corticosteroids. Dexamethasone was the most frequently studied agent, generally administered as a single dose of 4–10 mg by oral, sublingual, submucosal, intramuscular, intravenous, intra‐alveolar, intraosseous, or pterygomandibular routes. Prednisolone, methylprednisolone, and betamethasone were evaluated less frequently. In placebo‐ or control‐comparator trials, corticosteroids commonly reduced edema and limitation of mouth opening during the first postoperative days, although pain findings were not uniformly favorable. Betamethasone and some methylprednisolone protocols did not show clear advantages over placebo.

Route‐comparison trials generally suggested that clinically useful effects can be obtained through several routes. Oral and intravenous dexamethasone produced similar outcomes in one trial, intra‐alveolar and oral administration were comparable in another, and sublingual administration yielded lower pain and better mouth opening than intramuscular administration in one study. Because these trials used different doses, timing schedules, and surgical models, they do not establish a universally superior route.

Timing effects were also inconsistent. Preoperative methylprednisolone was more favorable than postoperative administration in one trial, whereas one submucosal dexamethasone study reported better pain control after postoperative injection. These findings indicate that timing may interact with the agent, dose, tissue site, and outcome rather than functioning as an independent determinant of effectiveness.

### 3.4. NSAID Trials

Eleven trials primarily evaluated NSAIDs, including ibuprofen, ibuprofen–arginine, meloxicam, etoricoxib, celecoxib, diclofenac, naproxen, oxaprozin, etodolac, nimesulide, and ketoprofen. Across placebo‐controlled and active‐comparator studies, NSAIDs were more consistently associated with postoperative analgesia and reduced rescue medication than with reductions in edema or trismus. Preoperative ibuprofen was not consistently superior to postoperative administration, and chronotherapy schedules did not materially alter pain or swelling in the small crossover study.

Within‐class comparisons suggested possible differences between individual agents or doses, but the findings were not uniform. Celecoxib and diclofenac provided better pain control than ibuprofen in one trial; etodolac reduced edema and trismus more than ibuprofen in another; and diclofenac produced less swelling than naproxen or etodolac at one postoperative time point. In contrast, nimesulide and ketoprofen showed broadly similar outcomes. These comparisons were generally small and used different endpoints and assessment schedules.

### 3.5. Photobiomodulation Trials

Seven trials assessed photobiomodulation using red or near‐infrared wavelengths, most commonly between 660 and 980 nm. Protocols differed in power, energy density, irradiation points, application site, timing, and number of sessions. Several sham‐controlled trials reported lower pain scores after active photobiomodulation, but the benefits for edema and trismus were inconsistent. Trials using combined intraoral and extraoral application or repeated sessions more often reported improvements across multiple outcomes, whereas single‐session or exclusively extraoral protocols tended to show analgesic effects without consistent reductions in swelling or trismus.

Because the photobiomodulation trials used substantially different dosimetric parameters and frequently did not report all variables required for reproducibility, the findings could not be pooled or translated into a single preferred protocol.

### 3.6. Direct Between‐Class and Combined Comparisons

Only a limited number of trials directly compared the different treatment classes. Three studies directly compared a corticosteroid with an NSAID or NSAID‐containing regimen. Dexamethasone was associated with better edema or mouth‐opening outcomes than diclofenac or etoricoxib at selected follow‐up points, but the small samples and nonuniform outcome measures prevent generalization to all corticosteroids and NSAIDs. Two additional trials assessed dexamethasone combined with etodolac or other NSAIDs; these combinations mainly reduced early pain and rescue medication use compared with monotherapy, without consistently demonstrating proportional benefits for edema or trismus.

One photobiomodulation study used nimesulide in both sides or treatment conditions, allowing evaluation of the incremental effect of active irradiation rather than a direct class‐level comparison. Overall, the evidence architecture did not provide a connected set of common comparators across all three modalities and therefore did not support a network meta‐analysis or a defensible treatment ranking.

### 3.7. Rescue Medication and Adverse Events

Reduced rescue analgesic consumption was reported in several corticosteroid and combination‐therapy trials and in selected NSAID studies. Reporting was less consistent in photobiomodulation trials. No serious intervention‐related adverse event was reported. Short‐course or single‐dose corticosteroid regimens were generally well tolerated, NSAID‐related events were mild and transient when reported, and photobiomodulation was not associated with clinically important harms. However, adverse‐event reporting was often limited and was not standardized across trials.

### 3.8. Feasibility of Quantitative Synthesis

Quantitative pooling was not considered methodologically appropriate. Trials differed in intervention dose and route, photobiomodulation parameters, comparator type, parallel vs. split‐mouth design, pain scales, edema measurement methods, time points, and reporting format. Several studies presented medians, graphical results, or significance tests without the means, standard deviations, paired correlations, or effect estimates required for reliable pooling. The small number of trials sharing an identical treatment contrast and follow‐up time further limited meta‐analysis.

## 4. Discussion

This review synthesized 36 randomized trials evaluating corticosteroids, NSAIDs, photobiomodulation, or combined strategies after third molar surgery. The evidence indicates that each modality may be useful for selected postoperative outcomes, but the original literature does not constitute a valid three‐way comparison. The evidence base was dominated by within‐class, dose, route, timing, placebo, or sham comparisons, and only a small number of trials directly compared different treatment classes. Accordingly, the present synthesis does not establish an overall treatment hierarchy.

Corticosteroids, particularly dexamethasone, were frequently associated with reductions in edema and trismus in trials with placebo, control, or active comparators. This pattern is biologically plausible because corticosteroids inhibit phospholipase A2 and thereby reduce several downstream inflammatory mediators. Nevertheless, the magnitude and consistency of benefit varied across agents, doses, routes, timing schedules, and outcomes. Negative or neutral findings with betamethasone and methylprednisolone demonstrate that the apparent benefits of dexamethasone should not be generalized to every corticosteroid regimen.

Route‐comparison studies did not identify a single administration route that was uniformly superior. Oral, sublingual, submucosal, intramuscular, intravenous, intraosseous, and intra‐alveolar approaches were all evaluated, often with different doses and surgical contexts. Although selected studies favored sublingual or submucosal delivery for particular outcomes, others found equivalent results across routes. Clinical choice should therefore consider feasibility, dose equivalence, patient preference, contraindications, and the specific outcome targeted rather than assuming a route‐independent class effect.

NSAIDs demonstrated their most consistent effects on postoperative pain and rescue analgesic use. Their influence on edema and trismus was less predictable, although individual trials favored diclofenac, etodolac, or intravenous ibuprofen for selected outcomes. These findings are compatible with cyclooxygenase inhibition as a strong analgesic mechanism but do not justify a uniform conclusion that NSAIDs lack anti‐inflammatory effects. Instead, the results suggest that drug selection, dose, route, and surgical trauma may modify the clinical response.

Photobiomodulation was associated with reduced pain in several sham‐controlled trials, whereas the effects on edema and trismus varied markedly. Protocol heterogeneity is a central explanation: wavelength, power, energy density, irradiation points, tissue depth, timing, and number of sessions differed substantially. Studies using repeated sessions or combined intraoral and extraoral irradiation more often reported multidimensional benefits, but this observation is based on indirect pattern recognition rather than formal comparative testing. Standardized dosimetry and complete reporting are necessary before a reproducible protocol can be recommended.

Direct evidence comparing treatment classes remained limited. Dexamethasone outperformed diclofenac‐ or etoricoxib‐based regimens for selected edema or mouth‐opening outcomes in a few small trials, and dexamethasone–NSAID combinations improved early analgesia in some studies. These findings are clinically relevant but should not be extrapolated to all agents within either class. Likewise, trials in which all participants received an NSAID while photobiomodulation was added evaluate an incremental effect and cannot be interpreted as a class‐level comparison between PBM and NSAIDs.

The inability to perform meta‐analysis was not based solely on statistical heterogeneity; the intervention contrasts themselves were often nonequivalent. Pooling different corticosteroid routes, distinct NSAIDs, or heterogeneous laser protocols at nonmatching time points could generate a precise but clinically uninterpretable estimate. The disconnected comparator structure also prevented a valid network meta‐analysis. A structured narrative synthesis was therefore the most defensible approach, provided that findings were separated by intervention and comparator type and that cross‐class ranking was avoided.

This review has several strengths, including prospective registration, inclusion restricted to randomized trials, duplicate study selection and data extraction, explicit risk‐of‐bias assessment, and organization of evidence according to the comparator architecture. Important limitations remain. Several trials had small samples, incomplete reporting of randomization or prespecified analyses, nonstandardized edema measurements, inconsistent follow‐up times, and inadequate numerical data for effect estimation. Adverse events were underreported, and the scarcity of directly comparable contrasts across intervention classes limited the strength of the comparative interpretation.

Future trials should prioritize direct head‐to‐head comparisons based on clinically relevant treatment questions, use standardized pain, edema, and mouth‐opening outcomes at common time points, report complete numerical data, register protocols prospectively, and describe photobiomodulation dosimetry in sufficient detail for replication. Such studies would permit conventional or network meta‐analysis and more reliable comparative recommendations.

## 5. Conclusion

Corticosteroids, NSAIDs, and photobiomodulation may each improve selected postoperative outcomes following third molar surgery. Corticosteroids were frequently associated with reductions in edema and trismus, NSAIDs showed more consistent analgesic effects, and photobiomodulation produced heterogeneous, protocol‐dependent findings. These observations describe patterns within the available comparisons and should not be interpreted as a definitive ranking among the three modalities.

The scarcity of direct head‐to‐head trials, the disconnected comparator network, and substantial variation in interventions, outcome measures, and follow‐up periods precluded meta‐analysis and reliable indirect comparison. Standardized randomized trials directly comparing clinically relevant regimens are required to determine their relative effectiveness and safety.

## Author Contributions

Érick Andres Alpaca Zevallos and Ilan Hudson Gomes de Santana conceived and designed the study. Érick Andres Alpaca Zevallos, Luciano Leocádio Teixeira Nogueira, and Anderson Jara Ferreira conducted the literature search, study selection, and data extraction. Camila Coelho Guimarães and Arthur Felipe de Brito Andrade contributed to data organization and methodological assessment. Ilan Hudson Gomes de Santana and Patrícia de Medeiros Loureiro Lopes performed the risk‐of‐bias assessment and critical appraisal. Elio Hitoshi Shinohara and Monique Danyelle Emiliano Batista Paiva contributed to the interpretation of the findings and scientific discussion. Ilan Hudson Gomes de Santana drafted the original manuscript.

## Funding

The authors received no specific funding for this study.

## Disclosure

All authors critically revised the manuscript, approved the final version, and agreed to be accountable for all aspects of the work.

## Ethics Statement

Not applicable. This systematic review used data from previously published studies and did not directly involve human participants or animals.

## Consent

The authors have nothing to report.

## Conflicts of Interest

The authors declare no conflicts of interest.

## Data Availability

All data analyzed during this systematic review were obtained from published studies cited in the manuscript. No new primary dataset was generated. The extracted study‐level data supporting the findings are presented in Table 2 and can be verified in the cited primary studies.
